# Fibronectin 1: A Potential Biomarker for Ovarian Cancer

**DOI:** 10.1155/2021/5561651

**Published:** 2021-05-22

**Authors:** Huijing Bao, Qianyu Huo, Qin Yuan, Chen Xu

**Affiliations:** ^1^The Integrative Medical Diagnosis Laboratory, The Tianjin Nankai Hospital, Tianjin, China 300100; ^2^School of Laboratory Science, Tianjin Medical University, Tianjin, China 300203; ^3^The Department of Laboratory Science, Tianjin 4th Central Hospital, Tianjin, China 300140

## Abstract

**Methods:**

OVCAR3 and A2780 are the two common cell lines that are used for ovarian cancer studies. The different invasion and migration abilities were observed by scratch tests and transwell experiments in our preliminary study. Gene chip was used to screen the expression gene in these two different cell lines, and then, the differentially expressed genes (at least 2-fold difference, *P* value < 0.05) were analyzed using KEGG.

**Result:**

Fibronectin 1 (FN1) was found to be the most strongly correlated with the invasion and migration abilities of the OVCAR3 cells. Real-time PCR and FN1 knockout cell line was conducted and confirmed this finding. Based on the Oncomine database analysis, comparing with normal people, ovarian cancer patients exhibited high levels of FN1 expression. Additionally, higher FN1 expression was found in patients with higher FIGO stages of cancer.

**Conclusion:**

FN1 could be a new biomarker for ovarian cancer detection and progress indicator.

## 1. Introduction

Ovarian cancer is the fifth leading cause of cancer-related deaths among women worldwide; unfortunately, early detection tests are relatively lacking. Furthermore, most women with ovarian cancer are diagnosed in the late stages of the disease, which portends a poor prognosis [1, 2]. Current surveillance strategies, which are achieved by transvaginal ultrasound or serum tumor marker cancer antigen 125 (CA125) tests, are ineffective in detecting ovarian cancer at an early stage [3, 4]. Therefore, the early detection and diagnosis of ovarian cancer have a very important role in the survival and prognoses of patients with epithelial ovarian cancer.

Fibronectin (FN) is a high-molecular-weight glycoprotein of the extracellular matrix that mediates a wide variety of cellular interactions with the extracellular matrix (ECM) and plays important roles in cell adhesion, migration, growth, and differentiation [5, 6]. FN1 is a member of the FN family, which has different functions during a variety of biological processes including cell adhesion, cell migration, and cytoskeletal organization in many different diseases [7]. In a variety of tumors, such as nasopharyngeal carcinoma, osteosarcoma, esophageal cancer, and ovarian cancer, FN1 is an important tumor-related gene [8–11].

Currently, the cell lines A2780 and OVCAR3 are commonly used in many different studies of ovarian cancer [12, 13], but no examinations of the differences between these two cell lines have been reported. In our study, greater migration and invasion abilities were found in the OVCAR3 cells compared with the A2780 cells. Therefore, we want to see if good protein candidates could be used as diagnostic markers for ovarian cancer based on these phenomena.

## 2. Materials and Methods

### 2.1. Cell Lines and Cell Culture

A2780, Caov3, and SKOV3 cells (CoBioer, China) were incubated in RPMI 1640 supplemented with 10% fetal bovine serum (Gibco, USA). OVCAR3 cells (CoBioer, China) were cultured in medium containing RPMI 1640, 10% fetal bovine serum, and 1% insulin (Sigma, China).

### 2.2. Cell Scratch Test

Cells were seeded into six-well plates, and when the density reached 100%, a scratch was made through the cells. Photographs were taken at 48 h, 72 h, or 96 h after the scratch using an inverted tissue culture microscope (Nikon ECLIPSE Ti-U) at ×4 magnification.

### 2.3. Transwell Migration Assay

The two ovarian cancer cell lines, i.e., A2780 and OVCAR3 (5 × 10^4^ cells/well), were seeded on transwell inserts (polycarbonate filters with 8 *μ*m pores, Costor) filled with medium. Medium (600 *μ*l) was added to the bottom chamber. After 48 h of culture at 37°C under humidified 5% CO_2_ in air, the cells that remained inside the upper chamber were gently and completely removed with a cotton swab. The cells that migrated to the lower surface of the filter membrane were fixed with anhydrous methanol and stained with 0.1% crystal violet. The filters were air dried, and photographs were collected with an inverted phase-contrast microscope (Nikon ECLIPSE Ti-U). After collecting the photographs, the membrane was washed with 500 *μ*l 50% aqueous acetic acid solution for approximately 5 min, 100 *μ*l of an eluent was added to a clean 96-well plate, and the OD values were measured at 595 nm using an ELISA reader (Synergy 2, China).

### 2.4. Microarray Analyses of the Nucleic Acids

Microarray analyses of the nucleic acids were performed using Affymetrix® Human Genome U219 Array Stripes (Affymetrix, USA) according to the manufacturer's instructions. The procedure included genomic DNA extraction fromA2780 or OVCAR3, digestion and ligation, PCR amplification, PCR product purification, quantification and fragmentation, labeling, array hybridization, washing, and scanning. Gene expression analyses were performed using the Affymetrix® Transcriptome Analysis Console (TCA) software, which evaluates the expressions of different genes. The Kyoto Encyclopedia of Genes and Genomes (KEGG) was also used for subsequent related pathway analysis.

### 2.5. Fluorescence Quantitative Real-Time PCR

RNA was isolated from each sample using TRIzol reagent (Ambion, USA) according to the manufacturer's instructions. First-strand cDNA was synthesized from the total RNA using oligo (dT) primers from the FastQuant RT Kit. Real-time PCR was performed on a stratagene MX3005P. Glyceraldehyde-3-phosphate dehydrogenase (GAPDH) was used as an internal control. The SYBR Green PCR Master Mix Reagent Kit was utilized for the PCRs, and the relative gene expressions were determined based on the threshold cycles (Ct) of the target gene and the internal reference gene. The average Ct value of the GAPDH gene was subtracted from the average Ct value of each target gene. The fold change (2^-*ΔΔ*Ct^) in expression was calculated for the gene of interest relative to the internal control gene (GAPDH) for each of the analyzed cancer cell lines. The primer sequences used in PCRs were as follows: FN1 sense: 5′-GTTCGGGAGGAGGTTGTTACC-3′ and antisense: 5′-GAGTCATCTGTAGGCTGGTTTAGG-3′ and GAPDH: sense: 5′-CCTCCAAGGAGTAAGACCCC-3′ and antisense: 5′-AGGGGTCTACATGGCAACTG-3′. Each sample was run in triplicate.

### 2.6. FN1 KO Cell Construction

293T cells were seeded on the plate, and then, the cells were cultured when the fusion degree was up to 80%. Lenti-CRISPR-V2-sgRNA-FN1 or lenti-CRISPR-V2, psPAX2, and pMD2.G were mixed into 1 ml Opti-MEM medium under a ratio of 4 : 3 : 1 and then incubated at room temperature for 5 min. 30 *μ*l PEI was added into the mixture, and then, the mixture was incubated at room temperature for 30 min. After incubation, the mixture was added to 293T cells and incubated at 37°C for 6 h with 5% CO_2_. After 48 h, the cell culture medium was changed to low serum medium (4% FBS), and the virus packaging supernatant was collected and stored at -80°C.

OVCAR3 cells were incubated for 24 h before virus transfection, and then, virus packaging supernatant with the final concentration of 10 *μ*g/ml polybrene mixed with complete medium under 1 : 1 ratio was performed into the OCVAR3 cells. After 36 hours of continuous culture, 0.2 *μ*g/ml puromycin was added into the medium for FN1 KO cell screening.

### 2.7. Western Blotting

Cells were washed in PBS and lysed in SDS loading buffer (KeyGEN BioTECH, KGP101) and subjected to the SDS-PAGE gel. The proteins extracted from the cells were transferred onto the PVDF membrane (Millipore, ISEQ00010). Membranes were blocked with 5% *w*/*v* milk (0.1%TBST) and incubated with mouse anti-FN1 antibody (affinity, BF0273, 1 : 1000) and mouse anti-beta-tubulin antibody (affinity, T0023, 1 : 2000) overnight in 4°C. Secondary antibody (affinity, S0002, 1 : 1000) was incubated with membrane for 1 h at room temperature.

### 2.8. ELISA

The experiment was conducted by the manuscript protocol (LiuHe, LH-E10073HU).

### 2.9. Statistical Analysis

SPSS software was used for statistical analysis. The results are expressed as the mean ± SD or the mean ± SEM. Two-tailed unpaired Student's *t*-tests were used to determine the statistical significance, and *P* < 0.05 was considered statistically significant.

## 3. Results

### 3.1. Detecting the Difference of Migration and Invasion Abilities

**Figure 1:**
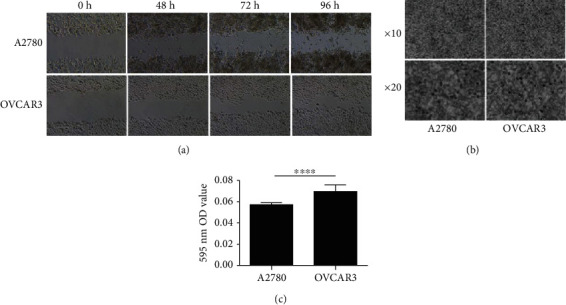
The migration capabilities of the A2780 and OVCAR3 cell lines were detected with scratch tests and transwell migration assays. (a) A wound healing assay was performed to assess the migration capacities of A2780 and OVCAR3 cells. (b) Transwell migration assays of the A2780 and OVCAR3 cells showing their migration abilities. (c) Quantitative results of the transwell invasion assays. Significant differences were identified using unpaired *t*-test analyses and are indicated by asterisks; ^∗∗∗∗^*P* < 0.0001.

To determine the differences of the migration and invasion abilities of these two ovarian cancer cell lines, scratch and transwell experiments were performed. The cells were seeded in 6-well plates, and the migration abilities were measured at 0 h, 48 h, 72 h, and 96 h. Compared with those in the A2780 group, the diameters of the scratches became narrow in the OVCAR3 group (Figure 1(a)). This difference indicated that the OVCAR3 cells exhibited stronger migration ability than A2780 cells. The invasion abilities were determined with the transwell experiments, and the numbers of invasive cells were counted at 48 h after the cells were seeded. The results revealed that a much greater number of invasive cells were observed in the OVCAR3 group (Figure 1(b)). This conclusion was also confirmed by the 595 nm OD reading results after the invasive cells were dissolved in 50% aqueous acetic acid solution (Figure 1(c)). Based on these results, the migration and invasion abilities of the OVCAR3 cells were much greater than those of the A2780 cells.

### 3.2. Determining the Different mRNA Expressions

**Figure 2:**
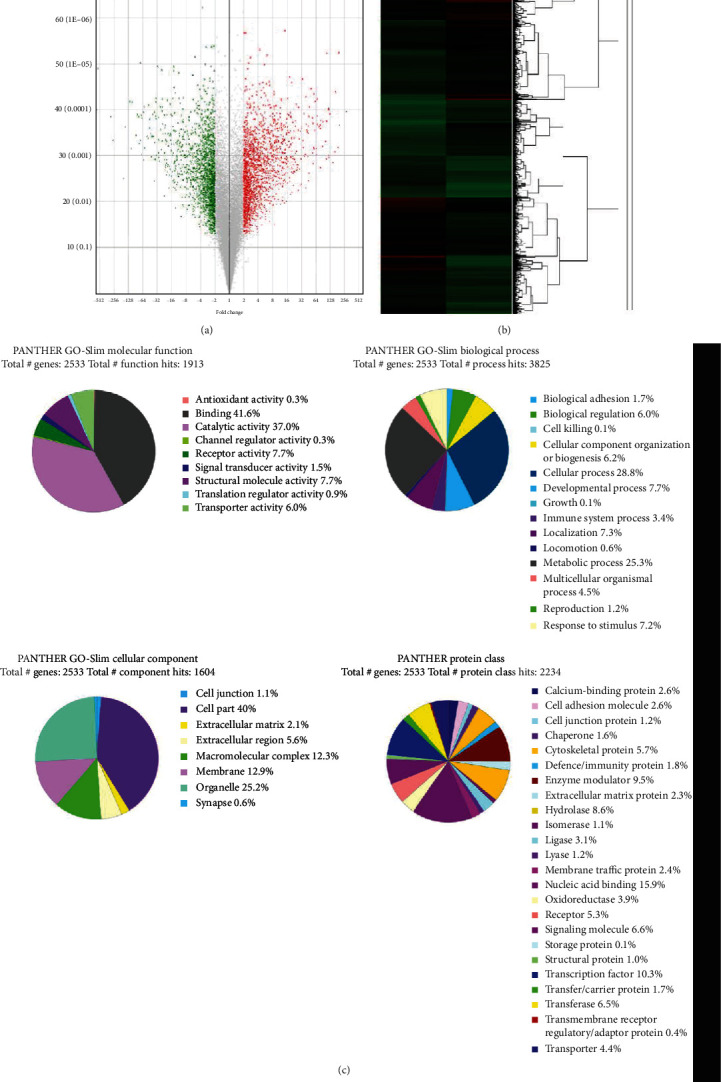
The gene expression profiles of the A2780 and OVCAR3 cell lines were analyzed with gene chips. (a) Volcano plot analyses showing the differentially expressed genes between the A2780 and OVCAR3 cells. The *x*-axis represents the log_2_ (fold change) value, and the *y*-axis represents the -log_10_*P* value. (b) Hierarchical clustering analysis of the differentially expressed genes. (c) Results of the gene ontology analysis. The percent followed by the category name is the percent of a gene hit against the total genes.

To determine the cause of the differences between these two cell lines, gene chip analysis was conducted with the OVCAR3 and A2780 cells. Many differentially expressed genes were found in these two ovarian cancer cell lines (Figures 2(a) and 2(b)) based on analyses of the volcano map results and the hierarchical clustering results. Additionally, the gene expressions of these two cell lines were analyzed in terms of the following four different gene ontology (GO) aspects: molecular function, biological processes, cellular components, and protein (Figure 2(c), continued). The results indicated that the two types of cells, i.e., A2780 and OVCAR3, exhibited significant differences both in gene expression and cellular biology.

### 3.3. Potential Protein Candidate Analysis

**Figure 3:**
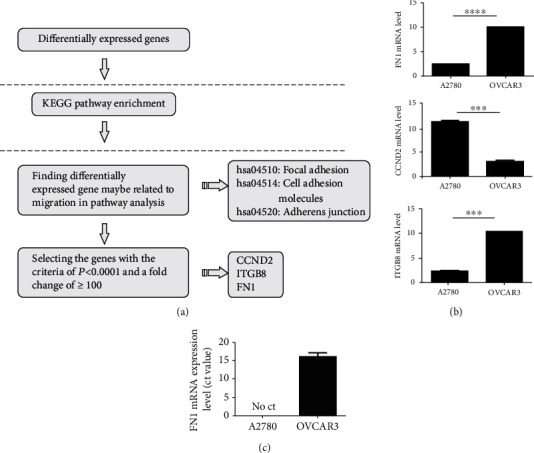
(a) Bioinformatics pipeline used to screen the candidate genes among the differentially expressed genes. (b) The three mRNA levels for FN1, CCND2, and ITGB8 on the gene chip. (c) RT-qPCR analyses of FN1 in the A2780 and OVCAR3 cells. GAPDH was used for normalization. Right, melting curves using the real-time PCR target genes (FN1, CCND2, and IGTB8) and the endogenous reference gene.

To identify different proteins, cell pathway analysis was performed using the Kyoto Encyclopedia of Genes and Genomes (KEGG). The differentially expressed genes between the two cell lines are presented in Table 1. The pathway with the greatest number of differences was the focal adhesion pathway. Moreover, some candidates genes (*P* < 0.0001 and fold change ≥ 100) among the focal adhesion-related genes (i.e., CCND2, ITGB8, and FN1) were selected for further analysis (Figure 3(a)). Statistical analyses of the expressions of these three genes revealed that the difference in FN1 expression was the most statistically significant (Figure 3(b)). To confirm the array results, real-time PCR was used to verify the difference in FN1 expression between the OVCAR3 and A2780 cells. The results revealed that the OVCAR3 cells did express FN1 at a higher level than the A2780 cells (Figure 3(c)), which proved that FN1 plays an important role in cell migration and that a high level of FN1 expression indicates high tumor cell mobility.

**Figure 4:**
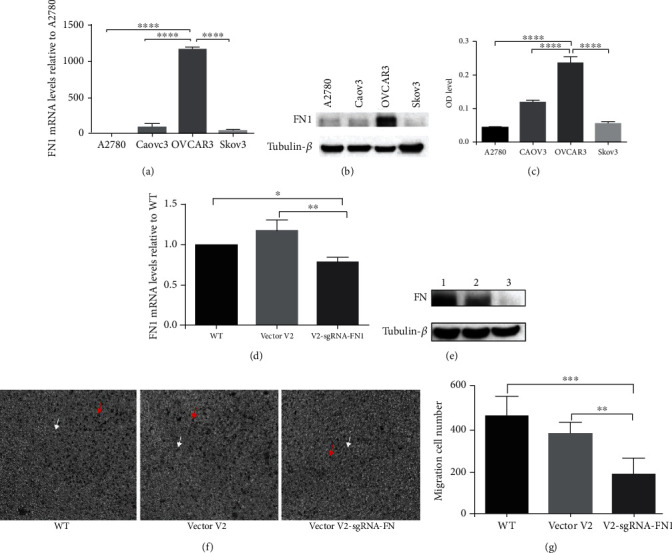
The FN1 KO cell was used for further confirmation. (a) The mRNA expression of FN1 was tested by real-time PCR among A2780, Caov3, OVCAR3, and Skov3. (b) The cell protein level of FN1 was detected by western blot among A2780, Caov3, OVCAR3, and Skov3. (c) The FN1 level in cell medium was detected by ELISA among A2780, Caov3, OVCAR3, and Skov3. (d) The knockout FN1 mRNA efficiency was detected by real-time PCR among wild-type cell (WT), control cell (vector V2), and the KO cell (V2-sgRNA-FN1). (e) The knockout FN1 protein level efficiency was detected by real-time PCR among wild-type cell (WT), control cell (vector V2), and the KO cell (V2-sgRNA-FN1). (f) Transwell assays were conducted among wild-type cell (WT), control cell (vector V2), and the KO cell (V2-sgRNA-FN1). (g) Quantitative results of the transwell invasion assays. Significant differences were identified using unpaired *t*-test analyses and are indicated by asterisks; ^∗∗∗^*P* < 0.001 and ^∗∗^*P* < 0.01.

To further confirm the results, FN1 KO cell line was constructed by CRISPR. Comparing the four different ovarian cancer cells, high FN1 expression was found in OVCAR3 at mRNA level (Figure 4(a)), protein level (Figure 4(b)), and the cell medium level (Figure 4(c)). So OVCAR3 FN1 KO cell line (V2-sgRNA-FN1) was conducted and confirmed at mRNA level (Figure 4(d)) and protein level (Figure 4(e)).

The differences in the migration and invasion abilities among the wild-type group, the control group (empty vector V2), and the KO group were detected by transwell experiments. The cells were seeded in 6-well plates, and the migration abilities were measured at 48 h. Compared with both the wild-type group and the control group, a significant less number of invasive cells were observed in the KO group (Figures 4(f) and 4(g)). Based on these results, FN1 should be a very important protein for the migration and invasion abilities of the ovarian cancer cells.

### 3.4. FN1 Clinical Feasibility Evaluation

Clinical samples were used for a FN1 feasibility evaluation. According to the above results, stronger invasion and migration abilities are associated with greater FN1 expression. This relationship indicates that FN1 expression could be used as a good indicator for disease progress.

**Figure 5:**
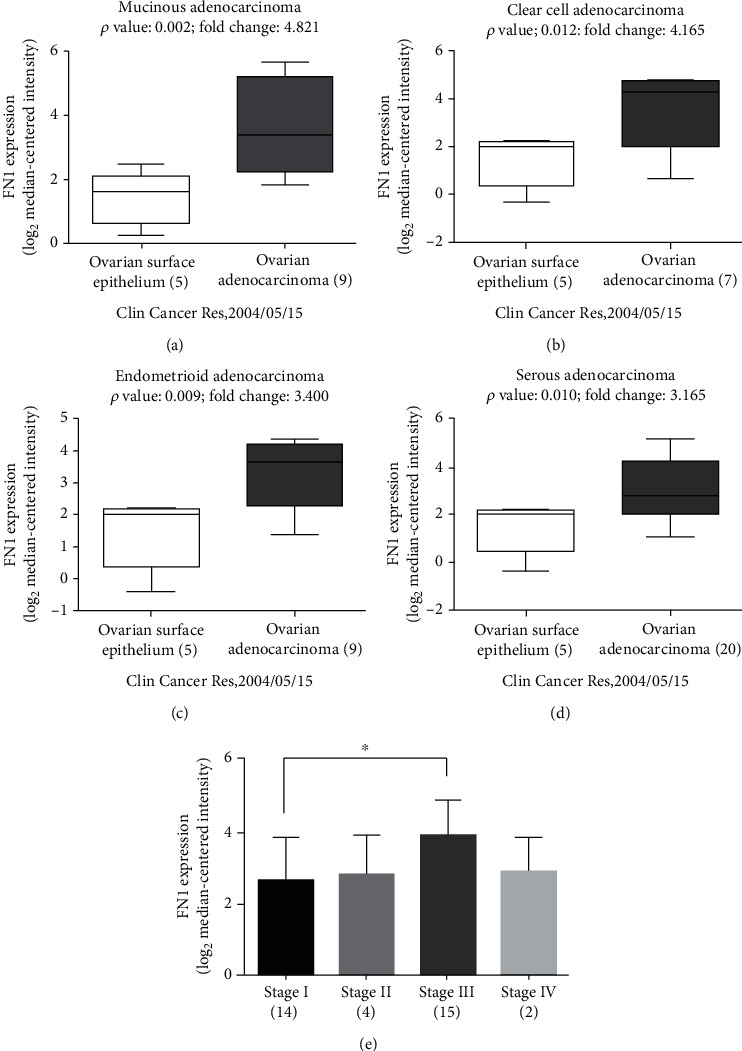
(a–d) Oncomine analyses showing that FN1 is highly expressed in human ovarian mucinous adenocarcinoma, ovarian clear cell adenocarcinoma, ovarian endometrioid adenocarcinoma, and ovarian serous adenocarcinoma. (e) Oncomine analyses showing the expression of FN1 in ovarian cancer according to the different FIGO stages. ^∗^*P* < 0.05.

To examine this possibility, the FN1 expression levels in patients with clinical ovarian cancer were identified using the Oncomine database with set conditions of *P* < 0.05 and a fold change of >3 or <-3. This analysis revealed that different pathological types of ovarian tissue exhibited different FN1 expression levels; ovarian mucinous adenocarcinomas (Figure 5(a)), ovarian clear cell adenocarcinomas (Figure 5(b)), ovarian endometrioid adenocarcinomas (Figure 5(c)), and ovarian serous adenocarcinomas (Figure 5(d)) exhibited higher FN1 expression levels than normal samples. The FN1 expression levels in patients in different FIGO stages were also analyzed. The results revealed that the FN1 expression levels in stage III were significantly greater than those in the low FIGO stages (stages I and II). This result indicated that FN1 expression is positively related to FIGO stage, i.e., greater FN1 expression is associated with a higher FIGO stage.

Taken together, our results indicated that FN1 could be used as a marker for aggressive ovarian cancer detection and can also be applied as an indicator of poor progression for the patients.

## 4. Discussion

Epithelial ovarian cancer (EOC) is one of the most common cancers among females and is the fifth leading cause of cancer death among women in the United States with 21,290 new cases and 14,180 deaths in 2015 [14]. Unfortunately, due to the lack of early detection markers and technical skills, most patients are diagnosed in the advanced stage of the disease, which is associated with a very poor survival rate and high levels of metastasis [15–17]. In our paper, we compared two different ovarian cancer cell lines with different migration and invasion abilities and found a new protein marker, FN1, which seems to be a strong candidate marker for the diagnosis of aggressive ovarian cancer. FN1 could also potentially be applied as an indicator of ovarian cancer progression or metastasis.

OVCAR3 and A2780 are the cell lines that are most commonly used in ovarian cancer studies [18–21]. In our laboratory, we found that these two different cell lines exhibit strongly different cell migration and invasion abilities. Transwell and scratch experiments were conducted to confirm these differences. The results revealed that the OVCAR3 cell line exhibited significantly stronger invasion and migration abilities than the A2780 cell line. To understand the different abilities of these two cell lines, gene chips were used to analyze the differences in gene expression. All are differentially expressed if they met the condition of a fold change greater than 2 or less than -2 with a *P* value < 0.05. To narrow the set of differentially expressed genes, stricter conditions were applied for the gene screening. After applying these stricter conditions (cell adhesion related, *P* < 0.001, and a fold change of greater than 100 or less than -100), only three genes (i.e., CCND2, ITGB8, and FN1) were found. After the expressions of the CCND2, ITGB8, and FN1 genes in the gene chip were statistically analyzed, the gene that was most strongly correlated with the invasion and migration abilities was found to be FN1. Next, real-time PCR experiments were used to confirm that the OVCAR3 cells did exhibit higher levels of FN1 expression than the A2780 cells, which exhibited weaker invasion ability than the OVCAR3 cells. And the FN1 KO cell line was used for further confirmation.

FN1 is a core component of many extracellular matrices where it regulates a variety of cell activities through direct interactions with cell surface integrin receptors. FN1 is synthesized by many adherent cells and then assembled into a fibrillation network [2]. Moreover, FN1 expression has been demonstrated to be closely associated with various migration processes, including wound healing, embryogenesis, and metastasis of cancer cells [22]. It has been reported that the extra domain A of FN1 could be a vascular marker of liver and lung metastases. Chen et al. found that both FN1 and TGM2 can facilitate the migration process of A431 tumor cells [23]. Another report also indicated that FN1 can significantly modulate the progression of glioma cells by preserving integrin *β*1 FN receptors in glioma cells [24]. Moreover, Franke et al. and Kujawa et al. also observed that fibronectin was an important prognostic factor in ovarian cancer and may be central to tumor progression [25, 26]. Based on these results, we believe that FN1 could be involved in the progression of ovarian cancer and could be the main reason for the differences in the migration and invasion abilities of these two cell lines. Actually, it has been reported that FN1 can prevent the apoptosis of ovarian cancer cells caused by therapeutic agents. It was suggested that FN1 could be used as a marker to indicate tumor progression in ovarian cancer. This hypothesis was confirmed by the clinical samples.

In our study, online database analysis (Oncomine) [10] was used to examine the expression levels in patients, and ovarian cancer patients were found to have higher FN1 expression levels than normal subjects. Additionally, there was a significant increase in FN1 expression in stage III cancer compared with lower FIGO stage cancers (stages I and II). Greater FN1 expression was associated with a higher FIGO stage. Both of the results indicated FN1 should be a good marker for ovarian cancer patients.

In conclusion, based on in vitro experiment results and the results from the online database analysis, we believe that FN1 could be used as a marker of ovarian cancer detection and could also be used as a progress indicator for ovarian cancer patients.

## Figures and Tables

**Table 1:** Pathway analysis of the differentially expressed genes (at least 2-fold change, *P* value < 0.05) using KEGG.

KEGG ID	Pathway name	*P* value
hsa04510	Focal adhesion	4.63*E* − 08
hsa04512	ECM-receptor interaction	2.77*E* − 07
hsa05206	MicroRNAs in cancer	1.98*E* − 05
hsa05412	Arrhythmogenic right ventricular cardiomyopathy (ARVC)	3.21*E* − 05
hsa05202	Transcriptional misregulation in cancer	7.83*E* − 05
hsa05134	Legionellosis	9.45*E* − 05
hsa04115	p53 signaling pathway	1.02*E* − 04
hsa04151	PI3K-Akt signaling pathway	4.32*E* − 04
hsa04360	Axon guidance	4.90*E* − 04
hsa04350	TGF-beta signaling pathway	6.73*E* − 04
hsa04670	Leukocyte transendothelial migration	8.52*E* − 04
hsa05146	Amoebiasis	0.001178
hsa05200	Pathways in cancer	0.001287
hsa04514	Cell adhesion molecules (CAMs)	0.001404
hsa05132	Salmonella infection	0.002408
hsa05222	Small cell lung cancer	0.002838
hsa04520	Adherens junction	0.003269
hsa05205	Proteoglycans in cancer	0.004471
hsa05203	Viral carcinogenesis	0.005616
hsa04310	Wnt signaling pathway	0.009113
hsa04550	Signaling pathways regulating pluripotency of stem cells	0.010103
hsa05220	Chronic myeloid leukemia	0.012857
hsa04062	Chemokine signaling pathway	0.013767
hsa04390	Hippo signaling pathway	0.017097
hsa05166	HTLV-I infection	0.018198
hsa04068	FoxO signaling pathway	0.019317
hsa04666	Fc gamma R-mediated phagocytosis	0.027802
hsa05120	Epithelial cell signaling in *Helicobacter pylori* infection	0.029704
hsa04668	TNF signaling pathway	0.032627
hsa05218	Melanoma	0.038013
hsa04932	Nonalcoholic fatty liver disease (NAFLD)	0.039736
hsa05164	Influenza A	0.042531
hsa04621	NOD-like receptor signaling pathway	0.04312
hsa04530	Tight junction	0.051741
hsa05219	Bladder cancer	0.054701
hsa04015	Rap1 signaling pathway	0.066471
hsa04810	Regulation of actin cytoskeleton	0.068376
hsa05142	Chagas disease (American trypanosomiasis)	0.072983
hsa04931	Insulin resistance	0.085386
hsa04540	Gap junction	0.088773
hsa04974	Protein digestion and absorption	0.088773
hsa04380	Osteoclast differentiation	0.089895
hsa00512	Mucin type O-glycan biosynthesis	0.093882
hsa04662	B cell receptor signaling pathway	0.094578
hsa05160	Hepatitis C	0.096068

## Data Availability

All the data has been presented in the paper.
